# Interaction of the Capsular Polysaccharide A from *Bacteroides fragilis* with DC-SIGN on Human Dendritic Cells is Necessary for Its Processing and Presentation to T Cells

**DOI:** 10.3389/fimmu.2013.00103

**Published:** 2013-05-06

**Authors:** Karien Bloem, Juan J. García-Vallejo, Ilona M. Vuist, Brian A. Cobb, Sandra J. van Vliet, Yvette van Kooyk

**Affiliations:** ^1^Department of Molecular Cell Biology and Immunology, VU University Medical CenterAmsterdam, Netherlands; ^2^Centre for Specialized Nutrition, Danone ResearchWageningen, Netherlands; ^3^Department of Pathology, Case Western Reserve University School of MedicineCleveland, OH, USA

**Keywords:** C-type lectin, dendritic cell, polysaccharide, pathogen, T cell proliferation

## Abstract

The zwitterionic capsular polysaccharide A (PSA) of *Bacteroides fragilis* is the first carbohydrate antigen described to be presented in major histocompatibility complex (MHC) class II for the induction of CD4^+^ T cell responses. However, the identity of the receptor mediating binding and internalization of PSA in antigen presenting cells remains elusive. C-type lectins are glycan-binding receptors known for their capacity to target ligands for antigen presentation to T cells. Here, we investigated whether C-type lectins were involved in the internalization of PSA and identified dendritic cell-specific intercellular adhesion molecule-3-grabbing non-integrin (DC-SIGN) as the main receptor for PSA on human dendritic cells (DC). The induction of PSA-specific T cell proliferation appeared to be completely dependent on DC-SIGN. These data reveal a crucial role for DC-SIGN in the endocytosis and routing of PSA in human DC for the efficient stimulation of PSA-specific CD4^+^ T cells.

## Introduction

In the defense against infection, specialized antigen presenting cells (APCs), such as dendritic cells (DCs) are able to endocytose pathogens into acidic compartments rich in endoproteases to allow the processing required for the extraction of antigenic epitopes. These peptide epitopes are then loaded onto major histocompatibility complex (MHC) class II and presented to CD4^+^ T cells. Until recently, peptides originating from the enzymatic degradation of protein antigens were thought to be the only class of molecules capable of being presented in MHC class II. However, these peptides can be posttranslationally modified with glycans, as has been shown for the MHC class II presentation of glycopeptides derived from the tumor antigen MUC1 (Vlad et al., 2002) or type II collagen (Michaëlsson et al., 1994).

Although DCs are able to endocytose different types of glycoconjugates and some of these glycoconjugates facilitate the induction of essential T cell help leading to the efficient production of glycan-specific antibodies (Astronomo and Burton, 2010); it was not until recently that the direct presentation of a polysaccharide on MHC class II was described. Zwitterionic polysaccharides (ZPSs) purified from the capsule of different bacteria were shown to activate CD4^+^ T cell responses without prior conjugation to a peptide (Tzianabos et al., 2000a,b). ZPSs are composed of repeating carbohydrate units in a polysaccharide structure, which contain both negatively and positively charged motifs, causing the formation of a helical structure (Choi et al., 2002), thereby resembling the structure of peptides in MHC class II. Removal of the positive charge in the ZPS of *B. fragilis*, polysaccharide A (PSA), led to the inability to fit into the MHC class II groove and thus failure to activate T cells. Strikingly, removal of the positive charge does not alter endocytosis, demonstrating that PSA is internalized independently of the charge of the molecule (Cobb and Kasper, 2008).

Protein antigens are normally processed by endoproteases in acidic compartments within the endo-lysosomal pathway. The short peptides generated are then loaded predominantly on MHC class II for presentation to CD4^+^ T cells. PSA also needs to be processed before it can be presented on MHC class II. We have previously shown that cleavage of PSA is a non-enzymatic oxidative reaction facilitated by the production of nitric oxide (NO) by inducible NO synthase (iNOS, EC.1.12.13.39), resulting in glycan structures of approximately 15 repeating units (Cobb et al., 2004). Furthermore, the formation of abscesses was completely absent in iNOS^−/−^ mice challenged with PSA or live *B. fragilis*, indicating that the iNOS-dependent degradation of PSA is a necessary event for presentation of PSA to T cells.

The mechanism of PSA internalization by DCs in order to enter the endo-lysosomal pathway for MHC class II presentation has remained unresolved until now. We suspected that since the internalization of PSA is a receptor-mediated event (Cobb et al., 2004) and DCs are equipped with a wide variety of antigen-uptake receptors including several C-type lectin receptors (CLRs) with specificity for glycans, CLRs would be likely candidates for the endocytosis of PSA. In addition, CLRs can target their ligands to the endo-lysosomal pathway for MHC class II presentation (van Vliet et al., 2008) and could therefore route PSA to MHC class II loading compartments. We here show that PSA is a ligand for the CLR dendritic cell-specific intercellular adhesion molecule-3-grabbing non-integrin (DC-SIGN) and that blocking DC-SIGN on DCs resulted in inhibition of PSA-specific T cell proliferation.

## Materials and Methods

### Fc chimeric proteins and antibodies

DC-SIGN-Fc is composed of the extracellular domains of DC-SIGN (amino acid residues 64–404) fused to the Fc domain of human IgG1 (Geijtenbeek et al., 2002). DC-SIGN-Fc was produced in CHO cells and purified with a Hi Trap Protein A HP column (GE Healthcare). The following antibodies were used: α-DC-SIGN [clone AZN-D1 (Geijtenbeek et al., 2000b)], α-dendritic cell immunoreceptor [DCIR, clone 111F8.04 (Dendritics)], and α-mannose receptor [MR, clone 19.2 (BD Bioscience)]. Stainings were performed using 5 μg/ml of primary antibody and 5 μg/ml of AlexaFluor 488 F(ab′)_2_ fragment of goat anti-mouse IgG (Invitrogen) as secondary antibody.

### PSA and Sp1

PSA was purified from *B. fragilis* as previously described (Cobb et al., 2004). Labeling was performed by oxidation of 20% of the galactofuranose side chain sugar. AlexaFluor 488 (AF488) was added via the aldehyde group (Cobb et al., 2004). Sp1 was prepared as described previously (Tzianabos et al., 2000b).

### Cells

Raji and Raji-DC-SIGN cells (Geijtenbeek et al., 2000a) were cultured in RPMI1640 (Invitrogen) supplemented with 10% FBS (BioWhittaker), 1000 U/ml penicillin/streptomycin (Lonza), and 2 mM glutamine (Lonza). Monocytes were isolated from PBMCs from buffy coats of healthy donors (Sanquin) by a lymphoprep gradient (Axis-Shield) and a subsequent percoll gradient centrifugation (Amersham). DCs were generated by culturing purified monocytes in RPMI1640 supplemented with 10% FBS, 1000 U/ml penicillin/streptomycin, and 2 mM glutamine in combination with IL-4 (262.5 U/ml; Biosource) and GM-CSF (112.5 U/ml; Biosource) for 4–7 days. CD4^+^ T cells were isolated from the PBL fraction of buffy coats of healthy donors via negative selection using MACS beads (Milteny Biotec) according to the manufacturer’s instructions.

### Binding/internalization assays

PSA-AF488 was incubated with DCs for 3 h at 37°C at the indicated concentrations in PBS containing calcium. For blocking experiments, 30 μg/ml PSA-AF488 was incubated with DCs for 45 min at 37°C in the presence of blocking agents and monitored by flow cytometry (FACScan, BD Biosciences). All flow cytometric analysis was performed with Flowjo software (Tree star). DC-SIGN expressing Raji cells were incubated with the indicated concentrations of PSA-AF488 for 3 h at 37°C. Unbound PSA was washed away and the amount of bound/internalized PSA was measured by flow cytometry. CLRs were blocked with a final concentration of 20 μg/ml of CLR-specific blocking antibodies, 50 mM monosaccharides [α-*N*-acetyl-d-glucosamine, α-l-Fucose, d-galactose, d-glucose, *N*-acetyl-d-glucosamine (Sigma Aldrich)], 25 μg/ml mannan (Sigma Aldrich), 25 μg/ml Laminarin (Sigma Aldrich), 25 μg/ml mannose-BSA (Sigma Aldrich), or 10 mM EGTA for 30 min at 37°C prior to the PSA binding assay.

### Internalization assay

PSA-AF488 was incubated with DCs for 3 h at 37°C at the indicated concentrations in PBS buffer containing calcium. As a positive and negative control, cells were incubated with a targeting antibody against DC-SIGN (AZN-D1) for 3 h at either 37 or 4°C respectively. Cells were washed in ice-cold PBS, fixated in 4% paraformaldehyde (Electron Microscopy Sciences), and measured by imaging flow cytometry (AMNIS Inc.) as previously described (García-Vallejo et al., 2012). Cells that have internalized antigen typically have positive internalization scores while cells that show the antigen still on the membrane have negative scores. Cells with scores around 0 have similar amounts of antigen on the membrane and in intracellular compartments.

### DC-SIGN-Fc binding assay

Ninety six-well flat-bottomed ELISA plates (Maxisorp, Nunc) were coated with 10 μg/ml or indicated concentrations of unlabeled PSA or Sp1 in PBS or Lewis^X^-polyacrylamide conjugate (PAA, Lectinity) in coating buffer (50 mM Na_2_CO_3_, pH 9.7) overnight at room temperature. DC-SIGN-Fc binding was measured as previously described (García-Vallejo et al., 2012).

### T cell proliferation assay

Irradiated (3500 rad) DCs were pre-incubated in the presence or absence of 20 μg/ml AZN-D1 for 30 min at 37°C. PSA was added at a concentration of 30 μg/ml and incubated for 3 h at 37°C. Unbound PSA and AZN-D1 were washed away and DCs were incubated with autologous CD4^+^ T cells for 6 days at a ratio of 1:10 in combination with 100 μM of the NO substrate glyco-SNAP-2 (Calbiochem). PSA binding and uptake and the blocking effect of α-DC-SIGN were monitored by simultaneously incubation of DCs with PSA-AF488 and measured by flow cytometry. Proliferation was measured using [^3^H]-thymidine incorporation (1 μCi/well; Amersham Bioscience) for 16 h. Cells were harvested onto filters and the [^3^H]-thymidine incorporation was measured with the use of a beta counter (Perkin Elmer).

### Statistical analysis

*P* values were calculated with a Student’s *t*-test. *P* values < 0.05 were considered to be significant.

## Results

### The interaction of PSA with DCs is carbohydrate-dependent

**Figure 1:**
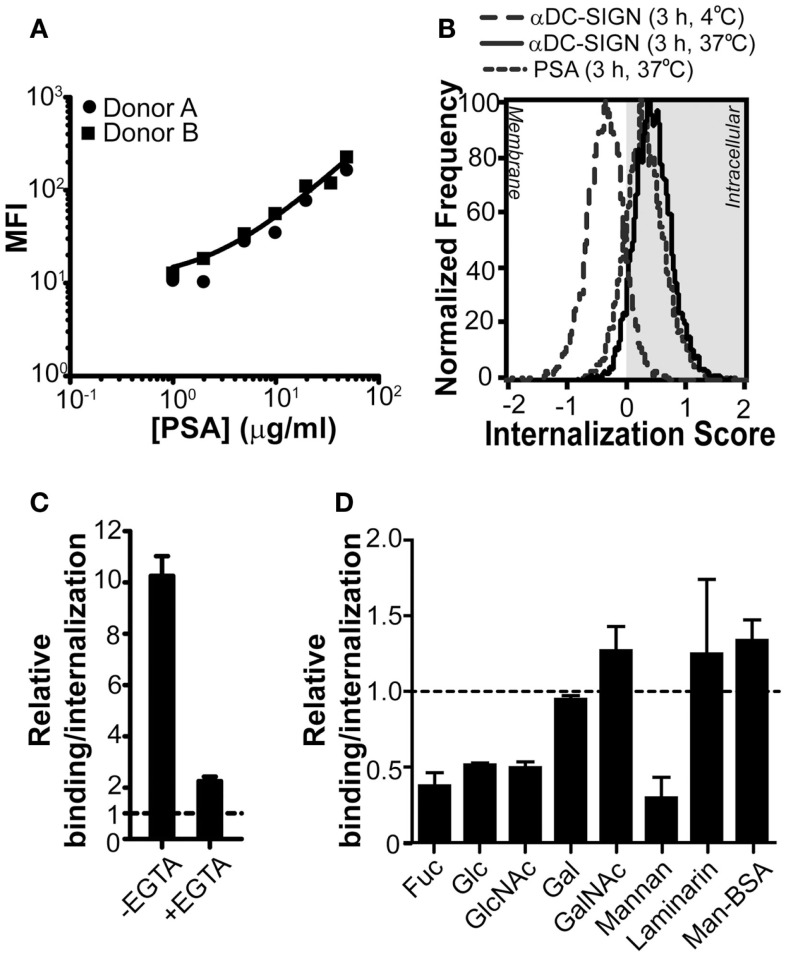
**PSA-binding/internalization by DCs can be blocked through Ca^2+^ chelation or by competition with glycans**. **(A)** PSA is recognized by DCs. PSA-AF488 binding was evaluated by flow cytometry after 3 h of incubation at 37°C. The line represents the average median fluorescence intensity (MFI) of the two donors. **(B)** PSA is internalized by DCs. Incubation of PSA-AF488 with DCs for 3 h at 37°C resulted in an efficient internalization of PSA. DCs incubated with a DC-SIGN-targeting antibody for 30 min at either 4 or 37°C were used as negative and positive controls, respectively. **(C)** PSA-binding/internalization is blocked by the addition of EGTA. Binding/internalization of PSA-AF488 by DCs was measured by flow cytometry in the presence of the Ca^2+^ chelator EGTA. Binding is shown relative to the autofluorescence of DCs. **(D)** PSA-binding/internalization is blocked by competition with mono and polysaccharides. GalNAc, α-*N*-acetyl-d-glucosamine; Fuc, α-l-fucose; Gal, d-galactose; Glc, d-glucose; GlcNAc, *N*-acetyl-d-glucosamine. Binding/internalization of PSA-AF488 by DCs was measured by flow cytometry after pre-incubation with the various mono and polysaccharides. Data is shown relative to DCs incubated with PSA in the absence of inhibitors. Data is shown as mean ± SD of a representative experiment out of six.

Although the unique feature of PSA to activate specific CD4^+^ T cell responses in humans has long been demonstrated (Tzianabos et al., 2000b), the molecular mechanisms leading to recognition and internalization, a crucial step in the processing and loading of PSA onto MHC class II, are still unclear. Since PSA is a complex carbohydrate and CLRs on DCs are involved in the recognition of glycans, CLRs appear to be the prime candidates for the recognition of PSA by APCs. In addition, several CLRs are antigen-uptake receptors for presentation on MHC class II or MHC class I (Sancho and Reis e Sousa, 2012). Therefore, we first analyzed whether DCs, known to express a wide variety of CLRs, were able to bind PSA. Our results show that human DCs efficiently bound PSA in a dose-dependent manner (Figure 1A). Although DCs exposed to PSA for a prolonged period of time at 37°C are expected to internalize any bound material, we could not deduce this from classical flow cytometry data. We therefore measured PSA-incubated DCs using imaging flow cytometry. As a positive and negative control we treated DCs with a monoclonal antibody against DC-SIGN, known to induce DC-SIGN internalization (Engering et al., 2002a), at 37 or 4°C, respectively. Triggering of DC-SIGN induced internalization only at 37°C. The signal corresponding to PSA perfectly overlapped with that of the positive control, indicating that PSA was efficiently internalized under these conditions (Figure 1B). To determine whether CLRs play a role in the binding/internalization of PSA we analyzed binding/internalization by flow cytometry in the presence of EGTA, a Ca^2+^ chelator. Results indicate that PSA binding/internalization by DCs was indeed Ca^2+^-dependent, a typical characteristic of CLRs (Figure 1C). In order to elucidate the identity of the CLR(s) involved in the recognition of PSA, we incubated DCs with PSA in the presence of different mono and polysaccharides as competitive inhibitors for CLRs. Binding/internalization of PSA could be blocked in the presence of fucose and mannan (Figure 1D), in concentrations previously shown to block DC-SIGN-dependent ligand binding (Curtis et al., 1992). In addition, high concentrations of glucose and *N*-acetyl-d-glucosamine (GlcNAc) could also block binding/internalization of PSA, although to a lesser extent (Curtis et al., 1992; Lee et al., 2011). Altogether these results indicate the involvement of a mannose/fucose-specific CLR, such as DC-SIGN or MR. Other mono and polysaccharides, like laminarin (a dectin-1-specific glycan) and GalNAc (a macrophage galactose-type lectin-specific sugar) did not alter PSA binding/internalization (Figure 1D). Also, Mannose-BSA (a MR-specific ligand) did not inhibit PSA binding/internalization (Figure 1D), suggesting that MR is not involved in the recognition of PSA by DCs. Therefore, we concluded that the CLR DC-SIGN is a likely PSA receptor on DCs.

### PSA interacts with DC-SIGN

**Figure 2:**
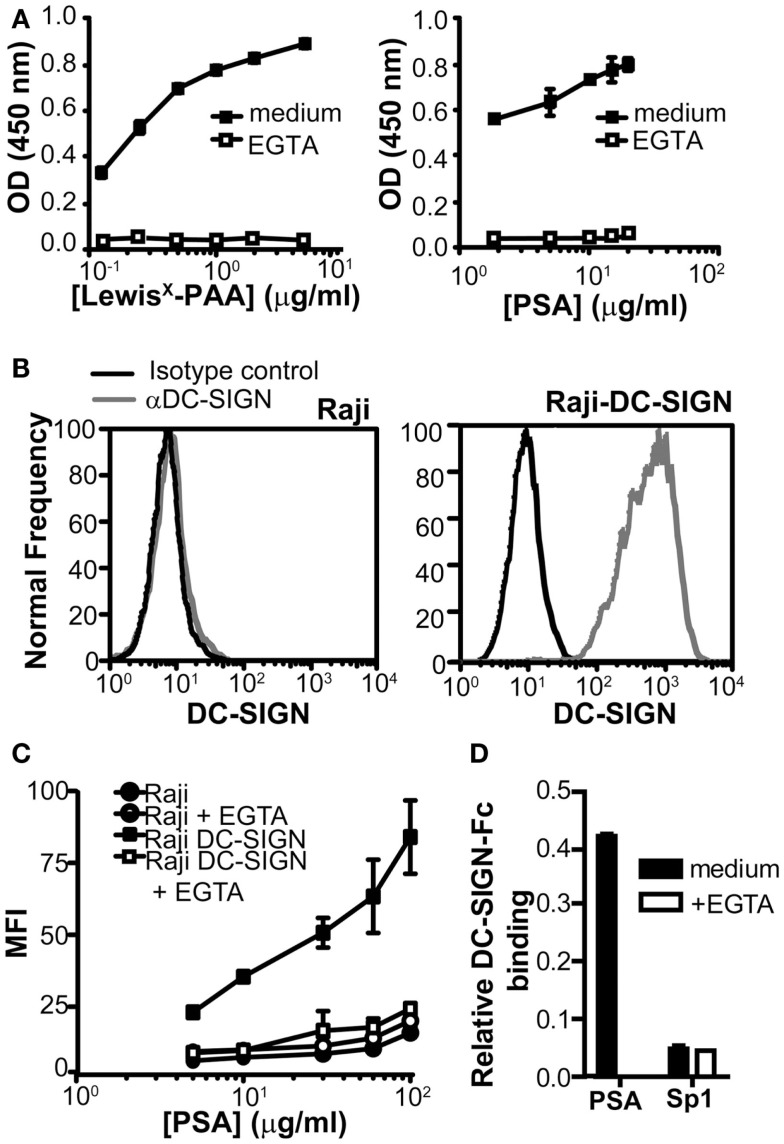
**PSA binds to DC-SIGN**. **(A)** Binding of PSA to DC-SIGN-Fc was measured using a DC-SIGN-binding assay. Binding of DC-SIGN-Fc to Lewis^X^-PAA served as a positive control. **(B)** Expression of DC-SIGN on parental Raji and Raji-DC-SIGN cells as measured by flow cytometry. Black line represents isotype control and red line indicates DC-SIGN expression. **(C)** PSA-binding/internalization via cellular DC-SIGN. Binding/internalization of different concentrations of PSA-AF488 was measured by flow cytometry after 3 h incubation at 37°C. CLR-specific binding was blocked by the addition of EGTA. **(D)** Sp1 is unable to bind the DC-SIGN-Fc construct. PSA and Sp1 were coated to an ELISA plate and DC-SIGN-binding was measured using a DC-SIGN-Fc binding assay. Data is shown relative to the non-coating control. Data is shown as mean ± SD of a representative experiment out of three.

To examine whether DC-SIGN directly binds to PSA, we used a DC-SIGN-Fc chimeric protein consisting of the extracellular domains, including the carbohydrate recognition domain of DC-SIGN fused to the human IgG1 Fc tail. We compared DC-SIGN-binding to PSA to the well-known DC-SIGN ligand Lewis^X^. Concentration-dependent binding of DC-SIGN-Fc to both PSA and Lewis^X^ was observed and the interaction appeared to be Ca^2+^-dependent, since it could be blocked by the addition of EGTA (Figure 2A). To confirm the binding of PSA at the cellular level, we used a Raji cell line transduced with DC-SIGN (Geijtenbeek et al., 2000a). Raji-DC-SIGN cells expressed high levels of DC-SIGN, which was absent on the parental Raji cells (Figure 2B). PSA bound the Raji-DC-SIGN cells in a concentration-dependent manner, which could be blocked by EGTA to the level of the parental Raji cells (Figure 2C). Several ZPSs have similar functional characteristics as PSA, including the induction of CD4^+^ T cell proliferation and the development of peritoneal abscesses (Velez et al., 2009). One of these ZPSs is Sp1, the capsular polysaccharide from type 1 *Streptococcus pneumoniae*. Although the biological properties of Sp1 mimic those of PSA, Sp1 was not recognized by DC-SIGN (Figure 2D), suggesting that DC-SIGN is a PSA-specific receptor and not a general binding partner for all ZPSs.

### Blocking DC-SIGN function inhibits PSA-induced T cell proliferation

**Figure 3:**
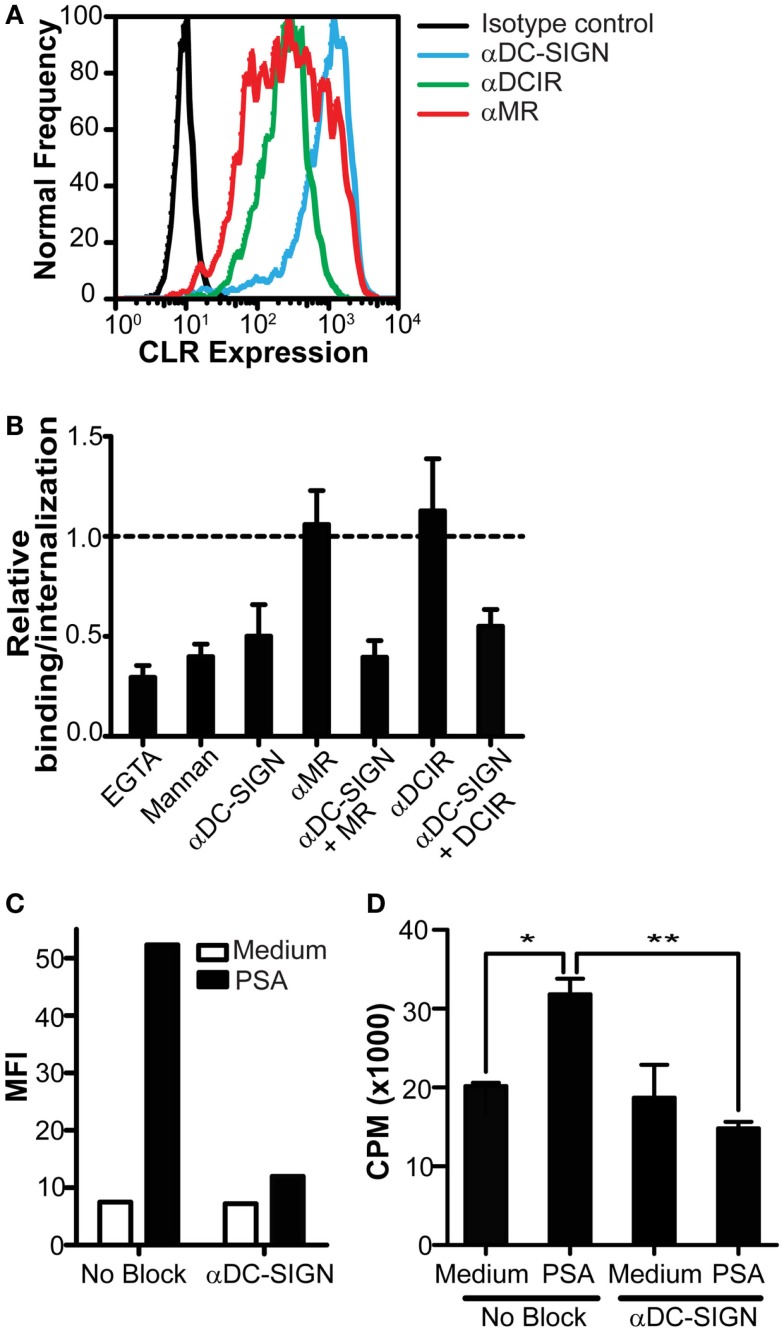
**Inhibition of DC-SIGN function decreases PSA internalization and PSA-induced T cell proliferation**. **(A)** Expression of DC-SIGN, MR, and DCIR on DCs as measured by flow cytometry. Black line represents isotype control, blue line indicates DC-SIGN expression, green line indicates DCIR expression, and red line represents MR expression. **(B)** PSA-binding/internalization by DCs is only blocked in the presence of a DC-SIGN blocking antibody. Data is shown relative to DCs incubated with PSA in the absence of inhibitors. **(C)** DC-SIGN blocking antibodies decrease PSA-binding/internalization by DCs. Binding/internalization of PSA-AF488 by irradiated DCs was measured by flow cytometry after 3 h incubation at 37°C. **(D)** Inhibition of DC-SIGN function decreases PSA-specific T cell proliferation. Irradiated DCs were pre-incubated with a DC-SIGN blocking antibody and subsequently incubated with PSA for 3 h at 37°C, extensively washed, and incubated with autologous CD4^+^ T cells in the presence of the NO donor glyco-SNAP-2. T cell proliferation was measured by [^3^H]-thymidine incorporation. Data is shown as mean ± SD of triplicates (*p < 0.05 ^**^p < 0.01). All experiments were performed three times with independent donors, one representative experiment is shown.

Since we identified DC-SIGN as a specific PSA receptor, we continued our research to determine the contribution of DC-SIGN in the presentation of PSA by DCs to CD4^+^ T cells. DCs express different CLRs, such as DCIR and MR, that share overlapping glycan specificity with DC-SIGN (Figure 3A). To rule out a possible involvement of these CLRs in the recognition of PSA, we tested the ability of different CLR blocking antibodies to inhibit PSA binding. Based on titration of the DC-SIGN antibody in previously published experiments (Geijtenbeek et al., 2000b), a concentration of 20 μg/ml blocking antibody was used. We observed that only the DC-SIGN blocking antibody decreased binding/internalization significantly, which was not further enhanced in combination with anti-MR or DCIR blocking antibodies (Figure 3B). These data confirmed DC-SIGN as the main receptor on DCs for the recognition of PSA. DC-SIGN functions as internalization receptor that mediates antigen routing to MHC class II loading compartments (Engering et al., 2002a). To assess the contribution of DC-SIGN in the MHC class II presentation of PSA to T cells, DCs were pulsed with PSA in the presence or absence of DC-SIGN blocking antibodies for 3 h, after which unbound PSA was washed away. PSA binding/uptake by DCs was clearly decreased in the presence of α-DC-SIGN (Figure 3C). The observed increase in CD4^+^ T cell proliferation by PSA-incubated DCs confirms the presence of PSA-specific T cells in blood (Figure 3D). Furthermore, PSA-specific T cell proliferation was completely inhibited in the presence of blocking antibodies to DC-SIGN (Figure 3D); demonstrating a DC-SIGN-dependent role in the uptake and processing of PSA for MHC class II presentation and subsequent T cell activation.

In conclusion, we have shown that DC-SIGN plays a crucial role in the binding and internalization of the T cell activating ZPSs PSA by human DCs. In addition, inhibiting DC-SIGN function decreases MHC class II presentation and subsequent T cell activation.

## Discussion

PSA is one of the ZPSs that can be presented in MHC class II for recognition by CD4^+^ T cells (Tzianabos et al., 2000b). Although internalization and NO-dependent processing of PSA is required for optimal presentation in MHC class II (Cobb et al., 2004; Duan et al., 2008), the identity of the receptor mediating binding and internalization of PSA has remained elusive. DC express several CLRs, specialized in glycan recognition and routing of internalized ligands to MHC class II or MHC class I loading compartments (Sancho and Reis e Sousa, 2012). We therefore hypothesized that a CLR might be involved and in this study we identified DC-SIGN as crucial receptor for PSA internalization and MHC-II presentation by human DCs.

Zwitterionic polysaccharides are generally composed of repeating oligosaccharide units. These oligosaccharide units are different for PSA and Sp1, explaining why PSA interacted with the purified DC-SIGN-Fc molecule, while the binding of Sp1 to DC-SIGN-Fc could not be detected. The oligosaccharide structure in PSA is [→3)-α-d-AAT Gal*p*-(1→4)-[β-d-Gal*f*-(1→3)] α-d-Gal*p*NAc-(1→3)-[4,6-pyruvate]-β-d-Gal*p*-(1→] (Tzianabos et al., 1992), while Sp1 is composed of the following oligosaccharide structure: [→3)-α-2,4-dideoxy-4-amino-d-FucNAc-(1→4)-α-d-GalA*p*-(1→3)-α-d-GalA*p*-(1→] (Velez et al., 2009). DC-SIGN has specificity for mannose- and fucose-containing glycan structures, and can therefore interact with a wide variety of pathogens (Appelmelk et al., 2003). However, both fucose and mannose glycans are not present in the PSA oligosaccharide. Since the β-d-Gal*f* glycan forms a side group in PSA, it is the most easily accessible carbohydrate for the binding to DC-SIGN. Sp1 from *S. pneumonia*, lacks this side group, potentially explaining the inability of DC-SIGN to bind to Sp1. It goes beyond the scope of this study to determine the exact glycan moiety in PSA that interacts with DC-SIGN. Together, our results indicate that DC-SIGN is a PSA-specific receptor and not a general binding partner for all ZPSs.

Next to the DC-SIGN-mediated PSA binding at the molecular level, we show an enhanced binding of PSA to DC-SIGN expressing Raji cells over the parental cell line. Raji cells are frequently used as model for PSA internalization and presentation in MHC class II (Kalka-Moll et al., 2002; Cobb et al., 2004; Cobb and Kasper, 2008; Duan et al., 2008; Ryan et al., 2011). In our assays PSA internalization was also detectable in the parental cell line, however this could only be observed when cells were incubated with high concentrations of PSA. DC-SIGN expression in Raji cells clearly increased PSA uptake and blocking DC-SIGN function on DCs significantly reduced PSA internalization and MHC-II presentation, while inhibition of other CLRs with overlapping glycan specificity, such as MR and DCIR, had no effect. These data indicate a crucial role of DC-SIGN in the binding and internalization of PSA by DCs.

DC-SIGN-binding ligands are quickly internalized and routed to endosomes and lysosomes (Engering et al., 2002a). Although the presence of DC-SIGN-binding glycans on protein antigens is necessary for the DC-SIGN-mediated routing to MHC class II loading compartments, it is a protein-derived peptide that is presented in MHC class II (Aarnoudse et al., 2008). Therefore, PSA is the first glycan structure internalized in a DC-SIGN-dependent manner that is presented solely onto MHC class II in the absence of a protein.

The presentation of PSA in MHC class II has previously been shown by the activation of human CD4^+^ T cell responses *in vitro* using PBMCs as APCs (Tzianabos et al., 2000b; Velez et al., 2009). Capture of PSA in this setting could be mediated by the small DC-SIGN^+^ subpopulation present in PBMCs (Engering et al., 2002b) or by the non-specific uptake typically observed in long term *in vitro* cell cultures using high PSA doses. DCs, on the other hand, are more likely candidates for the encounter of PSA under physiological conditions. We here demonstrate the ability of DCs to present DC-SIGN-internalized PSA in MHC class II, resulting in the activation of autologous CD4^+^ T cell responses. The induction of PSA-specific T cell proliferation could already be induced after short incubation times using relatively low concentrations of PSA, more closely mimicking the physiological setting. Activation of PSA-specific CD4^+^ T cells requires not only internalization of PSA, but also NO-dependent PSA processing and loading onto MHC class II (Cobb et al., 2004). Therefore, PSA loaded DCs were co-cultured with autologous CD4^+^ T cells in the presence of a NO donor (glyco-SNAP-2), to ensure the availability of sufficient NO to cleave the PSA. The use of glyco-SNAP-2 was necessary, since compared to DCs present *in vivo*, *in vitro* cultured monocyte-derived DCs hardly produce NO (Nishioka et al., 2003).

Besides being an internalization receptor, DC-SIGN has also been described to modify TLR signaling. Geijtenbeek et al. (2003) showed that binding of the mannosylated ligand ManLAM to DC-SIGN results in an increased IL-10 secretion by LPS-stimulated DCs. Surprisingly, the effect of DC-SIGN stimulation on TLR-induced cytokine responses is dependent on the interacting glycan. Whereas both mannose- and fucose-expressing ligands increase the IL-10 secretion by LPS-stimulated DCs, the effect on IL-12 and IL-6 secretion is different, whereby fucose-expressing ligands decrease the LPS-induced IL-12 and IL-6 production, while mannose-expressing ligands increase the LPS-induced IL-12 and IL-6 secretion (Gringhuis et al., 2009). The effects of PSA-mediated DC-SIGN stimulation still needs to be further investigated.

PSA-induced T cell proliferation has been reported in mice as well (Tzianabos and Kasper, 2002). In the murine genome eight different homologs of DC-SIGN are present (SIGNR1-8). The internalization capacity of these lectins has been investigated only for SIGNR1, SIGNR3, and SIGNR5, whereby SIGNR1 and SIGNR3 were reported to function as antigen-uptake receptors, whereas this was not the case for SIGNR5 (Takahara et al., 2004; Powlesland et al., 2006). SIGNR1 has been identified on lamina propria DCs in the murine intestine, where it mediates the induction of oral tolerance through the generation of Tr1 cells (Zhou et al., 2010). PSA derived from *B. fragilis*, a commensal bacterium in the gut, has also been described to induce regulatory T cells (Round and Mazmanian, 2010). Preliminary results from our laboratory indicate SIGNR1 as PSA-binding receptor (data not shown); however we cannot exclude the involvement of other receptors in the binding and internalization of PSA in mice *in vivo*.

In conclusion, we have shown that DC-SIGN plays a crucial role in the binding and internalization of the T cell activating ZPS PSA by human DCs. In addition, inhibiting DC-SIGN function decreases MHC class II presentation and subsequent T cell activation. These data indicate that DC-SIGN behaves as an antigen-uptake receptor for PSA, resulting in loading onto MHC class II molecules and the activation of PSA-specific CD4^+^ T cell responses.

## Conflict of Interest Statement

The authors declare that the research was conducted in the absence of any commercial or financial relationships that could be construed as a potential conflict of interest.
